# An Educational Initiative Describing Clinician Teachers’ Experiences Following Serious Illness Communication Skills Faculty Development Training

**DOI:** 10.1089/pmr.2024.0073

**Published:** 2025-05-26

**Authors:** Helen James, Jacquelin Forsey, Kyle Albuquerque-Boutilier, Jillian Gustin, Warren Harris Lewin

**Affiliations:** ^1^ Department of Supportive Care, Division of Palliative Care, University Health Network, Toronto, Canada.; ^2^ The Wilson Centre, University Health Network, Toronto, Canada.; ^3^ Department of Internal Medicine, Division of Palliative Medicine, The Ohio State University Wexner Medical Center, Columbus, Ohio, USA.; ^4^ Department of Family & Community Medicine, Division of Palliative Care, University of Toronto, Toronto, Canada.

**Keywords:** communication skills, faculty development, health professions education

## Abstract

**Background:**

Serious illness communication skills (SICS) are essential competencies for clinicians to possess. Unfortunately, SICS teaching is not routinely taught and many clinician teachers (CTs) never received training on how to teach them. We funded two cohorts of CTs to learn an evidence-based approach to SICS teaching to scale a unified approach to such training.

**Objective:**

The primary aim of this study was to explore CT experiences and attitudes toward SICS teaching following completion of VitalTalk Faculty Development training. Our secondary aim was to identify perceived barriers and enablers experienced by CTs in the translation of newly acquired skills into the workplace.

**Design/Measurements:**

Survey and semi-structured interviews administered post-training.

**Setting:**

Large metropolitan academic hospital in Canada. Fifteen (83%) of CTs completed the survey and 6 (40%) were interviewed. Participants were 38 years old (avg), female (80%), physicians (87%), nurse practitioners (13%), and in practice 8 years (avg).

**Results:**

One-hundred percent of participants would recommend the course to colleagues, believing it increased their SICS teaching quality and comfort to teach this topic in varied settings. Post-training, 90% of cohort 1 and 0% of cohort 2 taught SICS in workshops. Seventy percent of cohort 1 and 60% of cohort 2 taught SICS at the bedside across 10 specialties and interdisciplinary groups. Top cited teaching enablers were funding, protected time, and administrative support. All participants reported being likely to teach a workshop within the year. Eighty percent reported training increased their comfort to lead such conversations and 67% reported more frequently engaging in them. Qualitative analysis revealed that successful implementation requires SICS teaching to be valued at every level of the institution.

**Conclusion:**

Investing in faculty development for a group of CTs led to increased confidence in teaching about and having serious illness conversations and informed needs for a local community-of-practice primed to rapidly scale SICS teaching.

## Introduction

Serious illness communication skills (SICS) are essential components of high-quality health care and include disclosing serious news, responding to emotions, and recommending treatment aligned with patient goals and values. Research suggests serious illness conversations are associated with improved patient quality of life and reduced health expenditures.^1–3^ Residents are expected to develop SICS competency,^4–6^ yet such training is often insufficient and variable.^7–10^ Contributing to this variability is the lack of SICS training^11^ and faculty development (FD)^12^ available to support clinician teachers (CTs).

Training CTs to deliver effective SICS instruction ensures effective and consistent learning opportunities.^11^ FD enables the implementation of scalable and robust SICS training^12,13^ and clarifies training and practice standards. Evidence-based SICS programs have been developed to build these skills and prepare CTs to teach them.^14–17^ VitalTalk, an evidence-based SICS training program, is prevalent across North America, tied to a global community of practice (CoP), and backed by evidence.^18,19^ VitalTalk’s FD “train-the-trainer” course provides CTs with a framework to facilitate focused skills teaching in workshops and bedside coaching.

In Canada, serious illness care is provided by specialist palliative care (PC) teams, including physicians with one or two years of specialty training, or by non-PC physicians (e.g., family doctor and cardiologist) and their teams. While national postgraduate specialty-agnostic communication competencies exist, there remains no standardized national SICS curriculum, resulting in variable training experiences. At our institution, SICS training occurs during a mandatory PC rotation for family medicine trainees, but it is unknown if/how other programs provide SICS training. Prior local feedback indicates that trainees desire additional SICS opportunities outside of their PC rotation to experience learning through contextual variation.

Since 2020, members of our team have aligned on the need for an evidence-based approach to strengthen and unify SICS teaching. W.H.L. completed VitalTalk’s train-the-trainer course and taught SICS workshops locally with positive feedback. Concurrently, our department received philanthropic funding to scale SICS teaching within and beyond PC. To address this aim, we funded training for a group of CTs at our institution to build their SICS and their ability to teach them across specialties. CTs from teaching hospitals affiliated with our institution were nominated to attend the VitalTalk FD course due to previous involvement in, or expressed interest in SICS teaching, or reputation among local PC colleagues as strong communicators.

VitalTalk’s program was selected because: (1) its course provides a broad framework to teach any communication skill, making it adaptable across specialties and disciplines; (2) it was received positively by providers at our institution, and (3) it has demonstrated scalability in the United States. W.H.L. has trained, practiced, and taught on the topic in the United States and Canada. From these experiences, stakeholder discussions locally, and similar communication competencies between countries, we felt the FD course would not require cultural adaptations in Canada.

To our knowledge, no published studies describe post-VitalTalk-FD experiences. Our primary aim was to explore CT experiences and attitudes toward SICS teaching following completion of the course. Our secondary aim was identifying perceived barriers and enablers experienced by CTs in translating their newly acquired skills into practice.

## Methods

We employed a mixed methods approach to explore how the course and the local work environment interact to impact knowledge translation. After completing FD training, eligible participants were surveyed to gather demographic information and predominantly quantitative measures of their experiences. A smaller group of participants also completed semi-structured interviews to explore their experiences in richer detail.

### Participants

This project used a convenience sample limited by available philanthropic funding and to participants with adequate protected time to complete training. Eligible participants were CTs (physicians and nurse practitioners) at the University of Toronto who completed VitalTalk’s FD course.

### Faculty training

All CTs completed one three-hour virtual workshop taught by W.H.L. that introduced concrete skills to share prognostic information and respond to emotion empathically,^18^ and a structured approach to elicit values and goals to guide recommendations called REMAP.^20^ This workshop served as a prerequisite for VitalTalk’s FD course that comprised six full days of intensive training taught virtually by their faculty across two three-day sessions. Twelve CTs completed training in a course delivered exclusively for our institution, and six CTs completed their training in a course open to clinicians from varied institutions.

CTs completed the course during protected academic time. Approximately 95% of the course costs were covered through philanthropy. CTs paid $400 to secure their spot in the course and demonstrate commitment to training. During training, participants practiced SICS skills learnt during the prerequisite workshop while learning and practicing a structured approach to teaching them in workshop and bedside environments. The course aimed to help CTs feel comfortable teaching SICS using licensed VitalTalk materials at their local institution.

### Post-training teaching

CTs were encouraged to teach at least one workshop in the first year following their training to postgraduate trainees locally in their respective specialties. Funding was designated for resources to support the scaling of SICS teaching that included logistical support, and standardized patients for workshop training.

CTs were provided a standardized slide deck for didactic and workshop teaching that was adapted from VitalTalk by W.H.L. Cases and actor training for workshops were made available by W.H.L. CTs wishing to create unique cases were encouraged to do so using a provided template to minimize teaching variation.

### Data collection and analysis

#### Survey

**FIG. 1.**
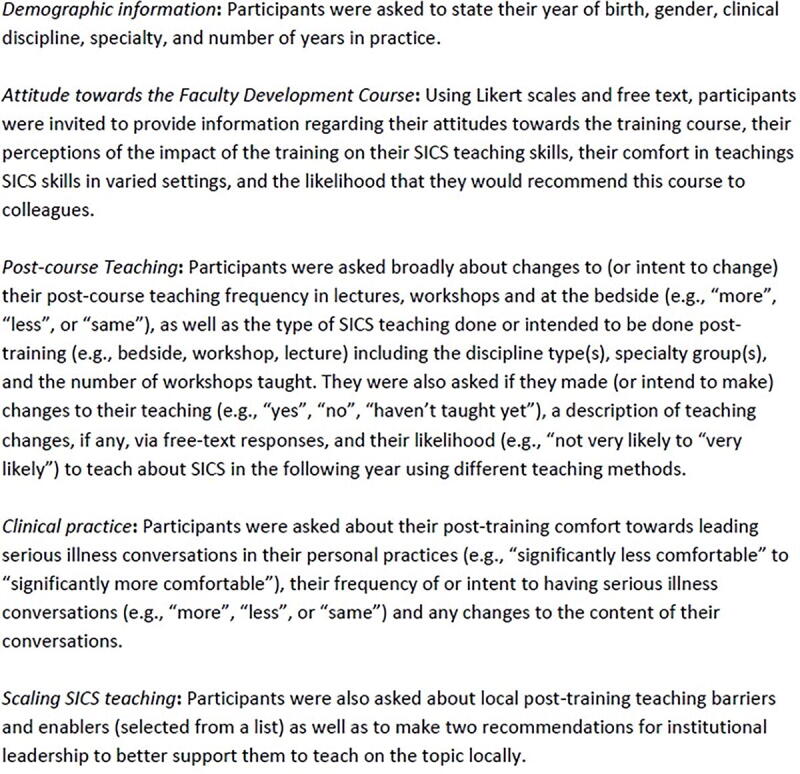
Faculty development survey description.

Participants were invited post-training to complete an anonymous online survey sent on behalf of the Principal Investigator and delivered electronically through Qualtrics^XM^. Surveys were disseminated approximately six months after the second cohort completed their training, and approximately one year after the first cohort completed their training. In the absence of an existing validated survey covering the areas of interest, a new survey was developed, guided by Kirkpatrick’s four-level evaluation model^21^ (e.g., reactions to training, new knowledge/skills, behavior changes) (Supplementary Appendix A). The survey is described in Figure 1.

#### Qualitative interviews

The team collaboratively developed an interview guide, which was refined iteratively by H.J. and W.H.L. after completing the first interview. The finalized guide (Supplementary Appendix B) was used by H.J. and K.A.-B. to conduct subsequent interviews. H.J. was a research fellow in our department who co-facilitated workshops and worked clinically with some participants. K.A.-B., a summer student, had no relationship to the participants. Interviews were 45–60 minutes long and audio-and-video recorded. Participants were asked to describe experiences facilitating SICS teaching, the impact of training on their teaching and clinical practices, perceived enablers and barriers impacting SICS teaching, and recommendations for leadership to enable future teaching.

Interviews were transcribed and de-identified prior to analysis. Transcripts were coded manually using NVivo. Qualitative analysis proceeded inductively and iteratively. The first round of open coding was conducted independently by H.J. and J.F. Subsequently, H.J., J.F., and W.H.L. met to create a refined codebook, which was then applied to the entire dataset. The jointly coded data underwent a process of increasingly finer categorization until all trends and variations were accounted for and cross-referenced. Final codes were grouped into categories and analyzed to understand broad themes capturing attitudes, perceptions, and experiences of participants.

Approval was provided by the University Health Network Research Ethics Board #23-5496.

## Results

### Survey

#### Demographic information

Of 18 eligible participants, 15 completed the survey (83%). Participant demographics are presented in Table 1. Forty percent (6/15) of participants were interviewed during four individual and one dyadic interview sessions.

**Table 1.** Demographic and Practice Characteristics

Demographic and practice characteristics	Total respondents (*n*, %)	Cohort 1 respondents (*n*, %)	Cohort 2 respondents (*n*, %)
Type of faculty development course	—	Exclusive to clinicians at our institution	Open to clinicians at any institution
Date of completion of faculty development course	—	September 2022 10/15 (66.7%)	June 2023 5/15 (33.3%)
Average year of birth (range)	1984 (1981–1989)	1984 (1976–1991)	1985 (1981–1989)
Gender			
Female	12/15 (80%)	8/10 (80%)	4/5 (80%)
Male	3/15 (20%)	2/10 (20%)	1/5 (20%)
Average years in practice	8.2 years Range, 1–19 years	8.6 years Range, 1–19 years	7.4 years Range, 2–13 years
Discipline			
Physician	13/15 (86.7%)	9/10 (90%)	4/5 (80%)
Nurse practitioner	2/15 (13.3%)	1/10 (10%)	1/5 (20%)
Specialties represented	Family medicine, critical care, neurosurgery, palliative care, internal medicine, and geriatrics		
Location of practice	Affiliated teaching hospitals from one university in a large metropolitan center in Canada		

#### Attitude toward the FD course

All 15 (100%) participants strongly agreed that they would recommend the training course to their SICS teaching colleagues and agreed that it gave them skills to be a better teacher. Most participants perceived the training increased the quality of their SICS teaching (workshop (93%), bedside (93%), and lecture (73%)), as well as their comfort teaching (workshop (93%), bedside (100%), and lecture (87%)).

#### Post-course teaching

Post-training, 90% (9/10) of cohort 1 participants taught SICS through workshops, 70% (7/10) through bedside teaching, and 60% (6/10) in lectures, to participants from nursing, social work, and physicians at varying levels of training and across ten specialties. Of participants who reported teaching workshops using standardized patients, three led four or more workshops, three led three workshops, and three led two workshops. Cohort 2 participants reported teaching less frequently: workshops (0%, 0/5), bedside teaching (60%, 3/5), and lecturing (40%, 2/5), but reported plans to train additional groups within the first year post-training, including in lung transplant, oncology, cardiology, and psychiatry programs.

Cohort 1 participants reported making changes to how they teach SICS: 70% (7/10) in bedside teaching, 80% (8/10) in workshops, and 20% (2/10) in lectures. Cohort 2 participants reported intending to change how they teach SICS: 100% (5/5) in bedside teaching, 100% (5/5) in workshops, and 80% (4/10) in lectures. Participants said: “[I] have more specific language to use to coach learners on communication skills at the bedside” and “[I’m] better able to orient learners to identifying the emotion and moving forward the conversation.” One-hundred percent (15/15) of participants reported being “very likely” or “likely” to teach SICS using a simulation workshop within the following year.

#### Clinical practice

Most participants reported the course also had positive impacts on their clinical practices. Eighty percent (12/15) reported increased comfort leading serious illness conversations with patients/families, and 67% (10/15) reported more frequently engaging in such conversations post-training. Participants said: “I spend more time on feelings and exploring values,” “I have better tools for responding to emotions and supporting families,” and “I have a better framework to follow.”

Our survey data showed that factors may be perceived simultaneously as teaching enablers and barriers, depending on their availability. Specifically, participants ranked time, funding, and administrative support to enable CT teaching opportunities, noting their absence as teaching barriers.

### Interviews

Themes derived from interview analysis are represented in Table 2 and described below:

**Table 2.** Interview Themes

Themes	Categories	Subcategories	Representative quote
Course content and structure	Teaching vocabulary and increased confidence	Language for teaching Increased comfort/confidence	“I think what was most useful is, first of all, that they give you a guide with exactly what they want you to say, so the prescribed language, that it’s done step by step.” (Participant 2)
	The value of structure	Bedside teaching Giving feedback Identifying learning opportunities Teaching method Standardization Structure for teaching	“I mean I think the brilliance of [workshop] is having people actually do it and say the words out loud in front of other people and get active feedback and try it again.” (Participant 1)
Community for collaboration and continued learning	Importance of a teaching community	Community of teachers Mentorship	“I think for the formal, I’ve been thinking about doing this for some time of going back and watching another skilled facilitator, or being an actor or something, or just being present to see how they stop and start things.” (Participant 2)
Enablers and barriers to knowledge translation in the workplace following faculty development training	Personal motivation	Personal Motivation	“Yeah, that’s a good point about the positive feedback. I do think that probably is definitely a motivator to keep going.” (Participant 5)
	Administrative and logistical support	Administrative support Teaching coordination/scheduling Resources	“I’d say the only thing maybe, and I don’t even know that this is logically possible, is it would have to be some central co-ordination of all of the teachings that are going on, in all the different sites, at all the different levels.” (Participant 6)
	Leadership recognition and support	Recognition for teaching Payment Funding Leadership Competing priorities for teachers	“Okay, well like in your annual review whether at the division level or with the university you could be praised and thanked for doing this. It could count towards promotion. I mean lots of things with regards to teaching and how undervalued it is.” (Participant 1)
	Clear expectations	Setting expectations Valuing communication skills teaching	“But if it was part of a strategic plan or vision, that communication skills were prioritized, then that would be a little bit top-down and then gradually, over time, if you had a champion within a division and then that number grew.” (Participant 4)

#### Course content and structure

Participants described the course having equipped them with vocabulary and a practical approach to skill-based teaching that helped them focus on discrete communication skills. Participants noted the training increased their confidence and comfort, and that receiving formalized training and certification empowered them to better define and establish their roles as SICS educators.

I have always felt that communication skills is a very important part of our job, but difficult to know how to teach … it just gave me tools and language and comfort and like courage I guess to actually teach it both in like formal teaching sessions, but also as opportunities came up within clinical supervision (Participant 1).

The structured nature of the SICS content and teaching approach helped participants feel effective and efficient with their teaching. The guidance on what and how to teach reduced perceived teaching burden. Some also felt that providing learners with a standard SICS language would improve teaching efficiency.

Time management is way better. I feel like it can almost be on autopilot, too. Like, the energy as a teacher feels very efficient in doing it (Participant 4).

While some participants felt more prepared to teach SICS at the bedside, other participants found it more challenging to do so. Barriers to bedside teaching were varied and included general (e.g., limited time, high stakes), structure specific (e.g., less adaptable to the fluctuating bedside environment), and course-specific barriers (e.g., less time spent practicing bedside teaching approach).

#### Community for collaboration and continued learning

Participants appreciated that the FD course helped build a local CoP that can act as peer mentors and champions. They identified practical needs to maximize future group success that included additional teaching-related education (including observing skilled facilitators, co-teaching, and refresher courses) and mentorship.

One of the things that I find really nice is I’m getting to know the other faculty members that are teaching and kind of developing a community around like-minded, like people that have the same interests (Participant 1).

Participants desire ongoing training opportunities to further hone their teaching skills and fill gaps in related topics that were not addressed during the FD course.

… to upskill us so that we can then have… a second-tier group who have already been through the training so that they can practice old skills, and maybe practice some new skills (Participant 6).

#### Enablers and barriers to knowledge translation in the workplace following FD training

Participants spoke about their precourse beliefs about the importance of SICS as personal motivation to seek teaching opportunities.

And so, I’m just so happy for the patients who now get a physician or nurse practitioner or social worker who may be just that much better of a skilled communicator, and that brings me immense joy (Participant 2).

They described having access to premade teaching materials, assistance with workshop coordination, and logistical support to significantly reduce the burden associated with organizing training. Participants felt that greater efforts to provide logistical coordination would enable continued delivery and expansion of SICS training.

Clear expectations for teaching post-training were considered beneficial by some participants. Additionally, participants identified that explicit learning expectations from institutional leadership would help focus training.

… to have expectations for teachers of, “we expect you to teach three to four courses a year,” will help to get the participation up (Participant 2).

Participants identified the role of leadership in shaping a culture where SICS are prioritized at the system, institutional, and discipline level. Participants felt this leadership support needed to be explicit and reinforced through practical resources. Moreover, participants felt that more recognition and rewards for SICS teaching from leadership and the organization would help incentivize teachers.

### Overarching and cross-cutting themes

The recurring theme across our analysis was that successful SICS FD required that these skills be valued and championed as essential medical competencies by academic institutions. This culture change is embodied by the individuals who practice SICS as well as the institutions that demonstrate their commitment to fostering SICS through their financial and administrative support. Participants expressed that their beliefs about the role of SICS teaching operated in tandem with the way that their institutions prioritize and/or reward teaching, to inform how they allocate time and resources.

## Discussion

Previously, it was unclear how formalized FD targeting SICS training might impact CTs who teach these skills at our institution. Our results demonstrated that the FD course offered CTs an evidence-informed approach to teaching SICS and providing high-quality feedback tailored to trainee learning goals. CTs reported feeling more skilled and comfortable teaching SICS post-training. Local administrative support for coordinating workshops, preprepared teaching resources, and clear expectations for post-training teaching positively contributed to perceived training impacts.

Training a group of CTs from one institution across multiple specialties brought together like-minded clinicians. While it was not our intention to create a CoP, given the outcomes observed and participant comments, it appears that more formalized support for the development and maintenance of a CoP was perceived as beneficial. CoPs have been created to foster a sense of belonging and connection, to share knowledge and build skill^22^ and are associated with collective growth, mentorship, and supportive learning opportunities.^23^ This type of social networking has also been shown to strengthen workplace teaching,^24^ which we are beginning to observe at our institution post-training.

Importantly, CTs reported that ongoing opportunities for skills practice and development post-training are essential elements of training implementation and for furthering skill development to better meet the teaching needs of advanced learners. Some CTs requested opportunities to observe or co-teach with more experienced CTs to build teaching confidence or further skill development. Future research should explore post-training CoPs to maximize the opportunities for FD and educational culture change.

The mix of participants from across specialties had the added benefit of breaking down silos and promoting a common language and approach to SICS training across our institution. This approach might offer learners consistent iterative observational learning opportunities in a variety of environments and medical contexts to consolidate learning. Further, it expands their SICS learning beyond PC, which underscores the importance of these conversations within all specialties. Moreover, having trained SICS teachers embedded in their own department’s culture might encourage peer buy-in and departmental leadership support. Further research should examine the impact of SICS role modeling by trained CTs on attitudes among peers who have not received such training.

Despite the benefits of this FD course, significant barriers to its sustainability were noted and consistent with what is known.^24^ Lack of time^25^ and administrative burden of training coordination hindered the CTs in implementing their newly acquired skills, and these same barriers have been linked to burnout among medical educators.^26,27^ To maximize post-training knowledge sharing, we argue that institutions should address the administrative burden that falls on medical educators through increased administrative support tied to FD knowledge translation plans (e.g., scheduling between CTs/trainees/standardized patients, booking teaching space, managing online learning, etc.).

Participants also noted that communication competency was rarely prioritized, influenced by organizational leadership in medical schools and hospitals. Medical educators often feel their role is undervalued and underrecognized,^28,29^ disincentivizing engagement in teaching. Opportunities for the CoP to interact and collaborate can amplify the group’s voice, fostering advocacy needed to influence organizational leadership in setting curricular priorities.

The study limitations include a small sample size from one institution and subjective retrospective responses to CTs teaching and clinical practices. Surveys were distributed at different post-training time points, which required modification of survey questions (see Supplementary Appendix A) and may have impacted CTs recall bias. Further, this study provided funding and dedicated time away from clinical work to support highly motivated CTs in the completion of FD training. Such support may not be available or feasible at other institutions.

## Conclusion

Investing in FD for a group of CTs motivated to teach about SICS led to increased confidence in SICS teaching and practice and implementation of skills in practice. Further, we identified the value of a local CoP that can be leveraged to collaborate across specialties to scale evidence-based and innovative approaches to SICS teaching, which can more rapidly disseminate knowledge on the topic across specialties broadly. CTs trained to use an aligned teaching approach were keen to collaborate to receive ongoing mentorship and training from their peers to maximize post-training knowledge that will contribute to closing the known training gap across specialties.

## Abbreviations Used

CoP: community of practice
CT: clinician teacher
FD: faculty development
PC: palliative care
SICS: serious illness communication skills
