# Supplementary material for: An Educational Initiative Describing Clinician Teachers’ Experiences Following Serious Illness Communication Skills Faculty Development Training
Source: Palliat Med Rep. 2025 May 26;6(1):291–8. doi: 10.1089/pmr.2024.0073 (PMC12410333; doi:10.1089/pmr.2024.0073)
Supplement: Supplementary Appendix A [file pmr.2024.0073_supplementary_appendix_a.pdf]

Appendix A. Faculty Development Post-Course Completion Survey

Survey

The following questions pertain to your VitalTalk Faculty Development course and your teaching experiences

Throughout this survey we will refer to 'serious illness communication skills.' In this context, this includes any skills that can be used within serious illness conversations with patients and their families (e.g., Headline, Wish statements, etc).

Serious illness conversations include discussion of patient values, sharing of prognosis, goals of care, advance care planning, breaking-bad-news, and family meeting conversations.

Demographic Questions

- In what year were you born? (Enter year)
    - ____ (YYYY)

- Gender identity: How do you describe yourself? (Select one response)
    - Male
    - Female
    - Do not identify as any of these options
    - Prefer not to answer

- How many years have you been in practice?
    - ____ (#)

- Select your discipline from the options below.
    a. Nurse Practitioner
    b. Physician

- Please indicate when you finished your VitalTalk faculty development course
    a. Sep 2022
    b. Jun 2023

Attitude towards the Faculty Development Course:

- Please indicate your level of agreement with the following statement: I would **recommend the** VitalTalk Faculty Development course to my clinical teaching colleagues who teach serious illness communication skills.
    a. Strongly disagree
    b. Disagree
    c. Neither disagree nor agree
    d. Agree
    e. Strongly agree

- Please indicate your level of agreement with the following statement: the VitalTalk Faculty Development course gave me **skills** to be a **better teacher**.
    a. Strongly disagree
    b. Disagree
    c. Neither disagree nor agree
    d. Agree
    e. Strongly agree

- Please indicate your level of **comfort** teaching serious illness communication skills using the following methods after completing the VitalTalk Faculty Development course.

	Significantly less comfortable	Less comfortable	Didn't change	More comfortable	Significantly more comfortable
Bedside teaching					
Simulation Workshop					
Lecture					
Other. Please describe <<free text>>					

- Please select how the VitalTalk Faculty Development course impacted the **quality** of your serious illness communication skills teaching using each of the following methods?

	Significantly lowered the quality	Lowered the quality	Didn't change the quality	Increased the quality	Significantly increased the quality
Bedside teaching					
Simulation Workshop					
Lecture					

Other. Please describe <<free text>>					

Post-Course Teaching:

- Would you say that you are teaching (or intend to teach) more, less, or the same amount of serious illness communication skills **at the bedside** since completing the VitalTalk Faculty Development course?
    a. More
    b. Less
    c. Same

- Would you say that you are teaching (or intend to teach) more, less, or the same amount of serious illness communication skills **in simulation workshops** since completing the VitalTalk Faculty Development course?
    a. More
    b. Less
    c. Same

- Would you say that you are teaching (or intend to teach) more, less, or the same amount of serious illness communication skills **in lectures** since completing the VitalTalk Faculty Development course?
    a. More
    b. Less
    c. Same

- Please select the methods of serious illness communication skills teaching you've done **since completing** the VitalTalk Faculty Development course **in which you've incorporated skills learned from the course**. Select all that apply.
    a. I have not used the skills in my teaching yet
    b. Bedside clinical teaching
    c. Simulation workshop with standardized patient (actor as patient)
    d. Simulation workshop without standardized patient (clinical team member as patient)
    e. Lecture
    f. Other: Please describe <<free text>>

    If selected c from above… "Was this simulation workshop a Powered By VitalTalk half-day workshop?"
    a. Yes
    b. No

If ‘yes’ to above... “How many Powered by VitalTalk half-day workshops have you facilitated or co-facilitated?”

i. 1
ii. 2
iii. 3
iv. 4+

- Please indicate which specialty or specialties you have taught (intend to teach) using skills learned from the VitalTalk Faculty Development course, either by bedside teaching, through a workshop, by lecture, or through other teaching methods. Select all that apply.
  a. Palliative Care
  b. Family Medicine
  c. Critical Care
  d. Internal Medicine
  e. Neurosurgery
  f. Cardiology
  g. Neurology
  h. Geriatrics
  i. Geriatric Oncology
  j. Hospitalist Medicine
  k. Oncology
  l. Other. Please indicate specialty below <<free text>>

- Select the types of healthcare providers you have taught (intend to teach) following your completion of the VitalTalk Faculty Development course. This could be bedside teaching, a workshop, a lecture, etc. Please select all that apply.
  a. Medical students (undergraduate)
  b. Medical residents/fellows (postgraduate)
  c. Medical staff
  d. Social workers
  e. Nurse Practitioner/Clinical Nurse Specialist
  f. Other. Please indicate below. << Free Text >>

- Have you changed Do you intend to change) how you teach about serious illness communication **at the bedside** since completing VitalTalk Faculty Development course?
  a. Yes
  b. No
  c. Haven’t taught at bedside yet *(this option only included for the second cohort)*

If ‘Yes’ to the above... Please tell us what changes you’ve made (or you intend to make) to your teaching? <<Free text>>

- Have you changed (Do you intend to change) how you teach about serious illness communication **in simulation workshops** since completing VitalTalk Faculty Development course?
    a. Yes
    b. No
    c. Haven't taught in a simulation workshop yet *(this option only included for the second cohort)*

  If 'Yes' to the above... Please tell us what changes you've made (you intend to make) to your teaching? <<free text>>

- Have you changed (Do you intend to change) how you teach about serious illness communication **in lectures** since completing VitalTalk Faculty Development course?
    a. Yes
    b. No
    c. Haven't lectured on this topic yet *(this option only included for the second cohort)*

  If 'Yes' to the above... Please tell us what changes you've made (you intend to make) to your teaching? <<free text>>

- Please tell us how **likely** you are to teach serious illness communication skills within the next year using the following methods.

	Very unlikely	Unlikely	Not Sure	Likely	Very likely
Bedside teaching					
Simulation Workshop					
Lecture					
Other. Please indicate teaching method. <<free text>>					

Clinical Practice

The following questions relate to your clinical approach to having serious illness conversations

- What is your personal level of comfort **leading** serious illness conversations with your own patients/families **since completed** the VitalTalk Faculty Development course?
    a. Significantly more comfortable
    b. More comfortable
    c. Same comfort level before and after
    d. Less comfortable
    e. Significantly less comfortable

- Did completing the VitalTalk Faculty Development course have any impact on how **frequently** you now address serious illness conversations with your own patients and families? Please select one.
    a. I engage in more frequent serious illness conversations
    b. I engage in the same amount of serious illness conversations
    c. I engage in less frequent serious illness conversations

- Did completing the VitalTalk Faculty Development course change the **content** of what you discuss when leading serious illness conversations with your own patients and families? Please select one.
    a. Yes
    b. No

    Please indicate what changes occurred. <<free text>>

Scaling teaching

- Enablers are structures or supports that can make something possible. Please pick the top three (3) enablers that would facilitate your ability to effectively teach serious illness communication.
    a. Protected time for trainees to learn and practice communication skills
    b. Protected time for faculty to teach
    c. Funding for standardized patients and/or stipend for teachers' time
    d. Administrative support (e.g. arranging and training SPs, booking rooms or managing calendar invites and Zoom, recruiting faculty facilitators, etc.)
    e. Mentorship or just-in-time coaching from an expert
    f. Other. Please detail below. <<free text>>

- Barriers are structures or obstacles that can prevent something from occurring. Please pick the top three (3) barriers that hinder your ability to effectively teach serious illness communication.
    a. Lack of funding (e.g., stipend) for teachers to be able to lead workshops
    b. Lack of funding for standardized patients and materials to lead workshops
    c. Lack of personal time to teach a half-day workshop
    d. Lack of support to coordinate and run workshops
    e. Lack of knowledge of what is needed to coordinate and run workshops
    f. Lack of interest from participants to attend workshops
    g. Lack of protected time for participants to join workshops
    h. Lack of ongoing faculty development training (e.g., skills refresher)
    i. Lack of mentorship or coaching opportunities for your facilitation skills
    j. Other. Please detail below. <<free text>>

- What 2 recommendations would you make to feel effective and be effective in implementing the Faculty Development skills in your teaching practice (e.g., what support would be helpful)?
  <<free-text response>>

Before you go….

- If there is anything else that you want to comment on, please feel free to write in the space below. <<Free text>>

  Thank you for completing this survey. If you have indicated you would like further information on participating in a focus group please look out for an email from our team in the coming weeks with a consent form to review, sign, and return.
