# Supplementary material for: An Educational Initiative Describing Clinician Teachers’ Experiences Following Serious Illness Communication Skills Faculty Development Training
Source: Palliat Med Rep. 2025 May 26;6(1):291–8. doi: 10.1089/pmr.2024.0073 (PMC12410333; doi:10.1089/pmr.2024.0073)
Supplement: Supplementary Appendix B [file pmr.2024.0073_supplementary_appendix_b.pdf]

Appendix B. Interview Questions for Focus Groups/Interviews

Order

- Informed consent (completed prior to session by participant signing consent form)
- Welcome
- Ground rules
- Start recorder
- Demographic questions
- Focus group discussion
- Stop recorder
- Thank you & incentives

Welcome:

Hello, my name is _____<<Facilitator name here>>_____. Thank you for agreeing to talk to me today. I am a research assistant working with The Conversation Lab completing **a study to understand how faculty felt about VitalTalk faculty development training, how they've implemented the skills learned in the course afterward, as well as enablers and barriers to teaching communication skills.**

As you know we conducted a survey of faculty that completed VitalTalk faculty development training. Thank you for completing that. We are now exploring faculty perceptions on this topic in more depth to determine how best to support teachers to teach about breaking bad news, ACP, GOC and related communication skills. I will refer to all these conversations as 'serious illness conversations'.

As a reminder, participation today is voluntary. We will be recording the interview to ensure we do not miss any information. Transcripts will be de-identified so that what you say cannot be traced back to you. The Principal Investigator will not have access to the focus groups and will not see any transcripts before de-identification.

During the interview, I will ask a series of questions. Please ask for clarification if anything is unclear. If you do not have anything to add or do not wish to answer, you can skip the question by telling me to do so. This session will take approximately 45-60 minutes. Do you have any questions before we go over the ground rules?

Ground rules:

- All voices are important, welcomed and your insights are valuable
- Please keep your phones/pagers off to be present for the conversation
- The recording will capture audio and video. If you are more comfortable keeping your camera off and changing your displayed name so identifying features are not captured in the recording please turn off your camera now.
- We'll follow 'Vegas rules' so please keep everything we talk about and the identity of the participants confidential

I'm going to start the recorder now and ask each of you for basic demographic information. Remember that everything you say will be de-identified so that your responses cannot be linked back to you.

Ask each participant the following:

- Preferred name
- Specialty you spend the most time working in (Neurosurgery, Palliative care, etc.)

Question Guide

IMPACT OF TRAINING ON TEACHING PRACTICE [10 minutes]

We are interested in hearing about how your thoughts on the VitalTalk faculty development course and how it impacted your teaching.

1. Faculty in the survey 'strongly agreed' that VitalTalk faculty development training gave them skills to be a better teacher. Can you describe how the training did this?
    a. Did anything about your Faculty Development training stand out? Tell me about what specifically stood out? Why did that stand out for you?
    b. Can you describe any **changes** to **how** you teach about serious illness communication since completing the faculty development course?
    c. Are there any changes to the **specific content** you teach?
    d. Are there any specific approaches or tools that you liked or found helpful from the course?
        i. What makes them helpful?
        ii. What do you think helps trainees learn during encounters when you use the Faculty Development skills you learned?
    e. How did the course compare to any previous Faculty Development serious illness communication skills training you received?
    f. Has the training impacted on how you teach skills other than serious illness communication?
2. (only if someone says it didn't make them a better teacher) If this doesn't resonate with you – could you expand on why you wouldn't recommend it

ENABLERS AND BARRIERS [15 minutes]

The main goal of the Faculty Development course is to help you feel comfortable to teach serious illness communication skills. We would like to hear about teaching **enablers and barriers** you've experienced since completing the course. For the next few questions, I'd like you to think about your recent bedside, workshop or lecture teaching. Think about the specific teaching framework and skills you learned in the Faculty Development course and how you've been able to use them or not since completing the course.

We'll start with **enablers**.

3. What has **enabled** you to use the tools/skills or to teach communication skills since completing your training?
    a. This could be UHN/institutional enablers, Conversation Lab enablers, department enablers or personal enablers, things you learned in VitalTalk faculty development training that enabled you to teach.

I'm going to switch gears now and ask you to think about any **barriers** to teaching communication skills.

4. Have you had any **roadblocks** or encountered **barriers** to using the tool/skills or teaching?
    a. Tell me about any specific barriers you've experienced?

BUILDING ORGANIZATIONAL CAPACITY [10 minutes]

Our goal is to have everyone that completed VitalTalk's faculty development course teach at the bedside and in workshops throughout the year to grow the number of UHN trainees and staff trained in evidence-based communication skills.

5. Can you think of any specific action(s) or support that would help you be successful to take the skills learned from the Faculty Development course and implement them into your teaching:
    a. Prompt: To help you teach at the **bedside**?

    b. What could UHN or leadership do to help you (or other people that are VitalTalk trained) teach at least **2-3 workshops** yearly?
        i. Prompt: are there things your departmental leadership could do?
        ii. Are there things the postgraduate leadership could do?

We also recognize that there are other great ways for people to learn about communication skills outside of workshops and we also know that not everyone can take VitalTalk's Faculty Development course...

6. So, if UHN could design the ideal course or 'way' to teach serious illness communication skills to its trainees and clinicians, what would your **top two recommendations** be to leadership to help us teach core communication skills to everyone at UHN?
    a. This can be pipe dream type ideas – aiming for the stars
    b. Prompt: to train all residents, fellows, interdisciplinary staff and physicians..
    c. Prompt: this doesn't have to include only workshops.... think about any method to help us teach these needed skills to our colleagues and trainees at UHN.

IMPACT OF TRAINING ON CLINICAL PRACTICE [5 minutes]

We're nearing the end of our time together today. Before we go, I have two more questions.

We talked about how faculty development training impacted your **teaching**. I am also interested in hearing about if and how VitalTalk faculty development training might have impacted your **personal clinical** practice.

7. Some survey respondents told us they feel more **comfortable** leading serious illness conversations with their patients/families after completing the course. Does this resonate with anyone? (Prompt: How so?)
    a. What specifically about the training led to this change?

8. Is there anything else that we didn't cover today that you would like to tell me about that I can bring back to the folks at The Conversation Lab?

Thank you again for making time today to chat with me. Your insights are incredibly valuable. I will stop the recording now.

We will be sending you a gift card as a small token of appreciation for your time and insights today. Please be on the lookout for an email with directions on how to access your gift card.

If you have any questions about our study or about today's experience, please email Helen at helen.james@uhn.ca or call Helen at 437 246 7356. (put email and number in the chat)

Thank you and have a nice rest of your day.
